# IL-8 confers resistance to EGFR inhibitors by inducing stem cell properties in lung cancer

**DOI:** 10.18632/oncotarget.3389

**Published:** 2015-03-18

**Authors:** Yi-Nan Liu, Tzu-Hua Chang, Meng-Feng Tsai, Shang-Gin Wu, Tzu-Hsiu Tsai, Hsuan-Yu Chen, Sung-Liang Yu, James Chih-Hsin Yang, Jin-Yuan Shih

**Affiliations:** ^1^ Department of Internal Medicine, National Taiwan University Hospital and College of Medicine, National Taiwan University, Taipei, Taiwan; ^2^ Department of Molecular Biotechnology, Dayeh University, Changhua, Taiwan; ^3^ Department of Internal Medicine, National Taiwan University Hospital, Yun-Lin Branch, Yun-Lin, Taiwan; ^4^ Institute of Statistical Science, Academia Sinica, Taipei, Taiwan; ^5^ Department of Clinical Laboratory Sciences and Medical Biotechnology, National Taiwan University, Taipei, Taiwan; ^6^ Department of Oncology, National Taiwan University Hospital, Taipei, Taiwan; ^7^ Graduate Institute of Oncology, Cancer Research Center, National Taiwan University, Taipei, Taiwan; ^8^ Graduate Institute of Clinical Medicine, National Taiwan University, Taipei, Taiwan

**Keywords:** IL-8, EGFR, gefitinib, resistance, stemness

## Abstract

Epidermal growth factor receptor (EGFR)-targeted strategy is limited by resistance. We identify the potential genes involved in EGFR TKI (tyrosine kinase inhibitor) resistance and study the therapeutic mechanism in the non-small cell lung cancers. Potential genes involved in resistance were examined by analyzing datasets from a pair of EGFR TKI-sensitive (PC9) and TKI-resistant cells (PC9/gef). Blood specimens from patients taking EGFR TKI as first-line treatment were used to examine the correlation between drug's efficacy and IL-8 level. The effects of IL-8 on gefitinib-induced apoptosis, stemness, and *in vivo* tumorigenicity were investigated using established cell lines. We identified IL-8 was up-regulated in gefitinib-resistant cells, and high plasma IL-8 level was correlated with shorter progression-free-survival time. IL-8 overexpression suppressed gefitinib-induced apoptosis in gefitinib-sensitive cells. By contrast, suppression of IL-8 enhanced gefitinib-induced cell death in gefitinib-resistant cells. IL-8 also increased stem-like characteristics including aldehyde dehydrogenase activity, expression of stemness-related genes, clonogenic activity, side-population, and *in vivo* tumorigenicity. Consistently, knockdown of IL-8 leads to loss of stem cell-like characteristics in gefitinib-resistant cells. Our study demonstrates an important role for IL-8, and suggests IL-8 is a potential therapeutic target for overcoming EGFR TKI resistance.

## INTRODUCTION

Lung cancer is the leading cause of cancer-related deaths worldwide [1]. Epidermal growth factor receptor (EGFR) tyrosine kinase inhibitors (TKIs) are successfully used in non-small cell lung cancer (NSCLC) patients harboring EGFR-activating mutations [2]. Despite early responses to EGFR-TKIs, cancers develop resistance after around 10 months of therapy. The EGFR-T790M secondary mutation and c-MET amplification contribute to the majority of acquired resistance [3]. However, there are still some resistant mechanisms are incompletely understood. To facilitate the development of effective therapies against NSCLC, we elucidate the EGFR TKI-resistance machinery underlying tumor progression.

We previously demonstrated that epithelial-mesenchymal-transition (EMT), a process in which epithelial cells lose cell polarity, confers resistance to gefitinib [4]. EMT is regulated by chemokines [5, 6]. CCL18, inducing EMT and chemoresistance, was shown to elevate in adenocarcinoma patients and correlated with poor survival [7]. The chemokine, CXCL1/2, whose release was triggered by chemotherapeutic agents, conferred resistance to doxorubicin [8]. Trastuzumab-resistant cells with an EMT phenotype were shown to secrete more IL-6 and be highly enriched with cancer stem cells (CSCs) [9]. CXCL12 evokes mobilization of lung cancer cells and confers a high capacity for self-renewal on these cells [10]. Blocking receptors for the chemokines CXCL1 and CXCL12 retards tumor growth, reduces invasion, eliminates CSCs, and restores drug sensitivity [10, 11]. These suggest that chemokines are associated with the features of CSC and treatment resistance.

Accumulating evidence demonstrates that CSCs are capable of driving tumorigenesis, and resistance to chemotherapeutics and TKIs [12–14]. CSCs were identified and purified in lung cancer using functional assays including aldehyde dehydrogenase (ALDH) activity and side population [15–17]. We previously demonstrated that gefitinib-resistant cells (PC9/gef) exhibited stem-like characteristics, such as high potential for sphere formation, expression of stemness-related genes, and ALDH activity [18]. We previously isolated a small fraction of ALDH-positive cells from gefitinib-sensitive PC9 cells. These ALDH-positive cells have higher expression of stem cell features and are more resistant to gefitinib, and chemotherapeutic agents compared with ALDH-negative PC9 cells [18]. Side population has been shown to be enriched for CSCs [17]. Dr. Shien *et al*. reported that exposure to high-concentration of gefitinib resulted in expansion of side population and EGFR TKI resistance [19]. Hence, it was suggested that the subset of side population cells in the heterogeneous cancer cell population was resistant to EGFR TKIs.

In this study, we investigated the role of chemokine in modulating cellular sensitivity to gefitinib in lung cancer cells. We found that IL-8 plays a role in the gefitinib-resistance machinery through regulation of the CSC population.

## RESULTS

### IL-8 was identified as a potential chemokine with EGFR-TKI resistance through genetic screens

**Figure 1:**
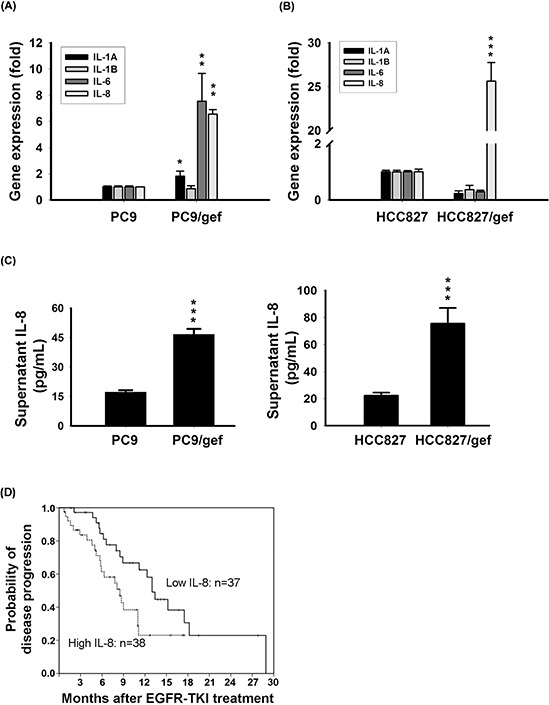
Up-regulation of IL-8 in gefitinib-resistant cells **A, B.** Expression of IL-8 mRNA was detected by RT-qPCR in PC9, PC9/gef, HCC827, and HCC827/gef cells. TBP (TATA-binding protein) was used as an internal control for normalization. The bar graph represents the mean ± s.d. for *n* = 3 independent experiments (***p < 0.001). **C.** IL-8 secretion by PC, PC9/gef, HCC827, and HCC827/gef cell lines was analyzed by ELISA. The bar graph represents the mean ± s.d. for *n* = 3 independent experiments (***p < 0.001). **D.** Kaplan-Meier survival curves of progression-free survival (PFS) after EGFR-TKI treatment in EGFR mutant lung adenocarcinoma patients with high (dashed) and low (solid line) plasma IL-8 expression (*p* = 0.02).

To investigate genes capable of conferring gefitinib-resistance in lung cancer cells, we selected a pair of cell lines- one gefitinib-sensitive (PC9) and one gefitinib-resistant (PC9/gef)- for analysis using oligonucleotide cDNA microarrays. An analysis of differentially expressed genes using the public DAVID bioinformatics resource was set up to identify the predominant molecular network listed in the Kyoto Encyclopedia of Genes and Genomes database (KEGG) (Supplementary Table S2). The differentially expressed genes of the predominant network- cytokine-cytokine receptor interaction included *IL-1*, *IL-6* and *IL-8* (Table 1). IL-1A, IL-1B, IL-6, and IL-8 are well-characterized cytokines involved in inflammation or chemoresistance [21]. We examined expression of *IL-1A*, *IL-1B*, *IL-6* and *IL-8* in two pairs of gefitinib-sensitive (PC9, and HCC827) and gefitinib-resistant (PC9/gef, and HCC827/gef) lung cancer cell lines to identify the specific cytokine involved in gefitinib resistance by RT-qPCR. We showed that *IL-1A*, *IL-6*, and *IL-8* were up-regulated in PC9/gef, but only *IL-8* mRNA was up-regulated in HCC827/gef (Fig. 1a–b). IL-8 protein was significantly elevated in PC9/gef and HCC827/gef (Fig. 1c).

**Table 1:** Cytokine and chemokine genes differentially expressed between PC9/gef and PC9 cells

Gene	Fold change (PC9/gef *vs.* PC9)
IL1A	74.62
IL1B	20.58
IL1F7	7.33
IL11	5.61
TNFRSF11B	4.30
CXCL6	3.79
IL8	3.38
CXCL1	3.13
IL6R	2.85
LTB	2.73
CXCL2	2.71
IL6	2.60
IL15	0.48
IL1RL1	0.41
IL20RA	0.41
TNFRSF19	0.33
TNFRSF1B	0.32
TNFSF10	0.26

Studied has reported that IL-8 is elevated in the plasma of cancer patients, and IL-8 is associated with poor prognosis and resistance to chemotherapy [22, 23]. Accordingly, we investigated whether IL-8 was involved in gefitinib resistance. Besides IL-8, IL-8-specific receptors, *CXCR1*, was significantly up-regulated in PC9/gef cells (Supplementary Fig. S1a). *CXCR1* is undetectable, but *CXCR2* was up-regulated in HCC827/gef cells (Supplementary Fig. S1b). We suggested that IL-8-CXCR1/2 signaling was involved in EGFR TKI resistance.

### High plasma IL-8 level revealed a shorter progression-free-survival of EGFR TKI-treated EGFR-mutation positive lung adenocarcinoma patients

To investigate the association of IL-8 levels with EGFR TKIs responsiveness, we collected peripheral blood samples from 75 stage IV lung adenocarcinoma patients with EGFR-mutation positive tumors and receiving EGFR-TKIs only as the first-line treatment. The EGFR mutation status of these patients was summarized in Supplementary Table S3. Of the 75 patients, 66 received gefitinib and nine received erlotinib. According to the median plasma IL-8 level (6.74 pg/mL), we divided patients into high-IL-8 and low-IL-8 groups. There were no significant differences in the clinical characteristics of high and low IL-8 groups (Table 2). However, median progression-free survival was longer in the low IL-8 group (13 months) than in the high IL-8 group (8.5 months; *p* = 0.02; Fig. 1d).

**Table 2:** Clinical characteristics of the 75 advanced lung adenocarcinoma patients who received EGFR-TKI as the first line treatment

	Patient No.	IL-8^1^		*P*
		High-expression	Low-expression	
**Total No.**	75	38	37	
**Age, mean years (range)**	63.9 (42.7–88.8)	65.1 (42.7–88.8)	62.7 (44.2–84.7)	0.408*
**Sex**				0.862^§^
** Female**	52	26	26	
** Male**	23	12	11	
**Smoking**				0.781^§^
** Nonsmokers**	64	32	32	
** Former/currentsmokers**	11	6	5	
**EGFR-TKI**				0.480^§^
** gefitinib**	66	32	34	
** erlotinib**	9	6	3	

1 Median IL-8 value: 6.74 pg/mL

* by Student *T* test

§ by Fisher Exact test

### IL-8 conferred resistance to EGFR TKI

**Figure 2:**
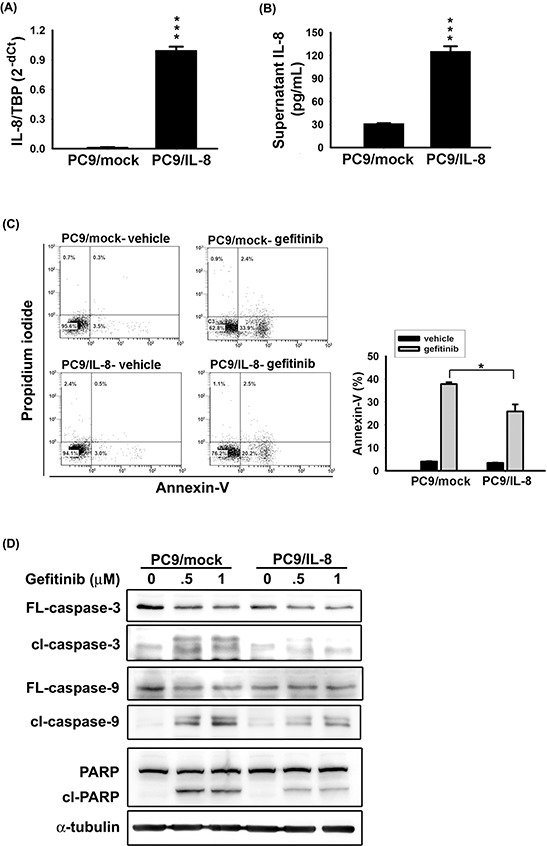
IL-8 conferred EGFR TKI resistance IL-8 expression in stable PC9/mock and PC9/IL-8 cell lines was evaluated by RT-qPCR **A.** and IL-8 ELISA **B.**. **C.** After 24 hours of treatment with 50 nM gefitinib, the percentage of apoptotic cells was evaluated by Annexin-V staining. The bar graph represents the mean ± s.d. for *n* = 3 independent experiments (*p < 0.05). **D.** The effect of IL-8 on gefitinib-induced apoptosis was evaluated by analyzing PC9/mock and PC9/IL-8 whole-cell extracts collected after 24 hour treatment with gefitinib (0.5 or 1 μM) for caspase-3, caspase-9, and PARP by Western blotting; α-tubulin was used as a loading control. Data are representative of three independent experiments.

To examine the role of IL-8 in the resistance to EGFR TKI, we established an IL-8-expressing PC9 cell line (PC9/IL-8). PC9/IL-8 expressed higher levels of *IL-8* mRNA and protein than the control cells (PC9/mock) (Fig. 2a–b). Increased Akt phosphorylation, NF-κB p50 nuclear translocation, and higher invasion ability in PC9/IL-8 suggest effective activation of IL-8 pathway (Supplementary Fig. S2).

The percentage of apoptotic cells, quantified by Annexin-V-positive cells, significantly decreased in PC9/IL-8 than in PC9/mock following exposure to gefitinib (Fig. 2c). Furthermore, treatment with gefitinib clearly induced cleavage of caspase-3, caspase-9, and poly-(ADP-ribose) polymerase (PARP) in PC9/mock (Fig. 2d). In contrast, activation of these pro-apoptotic proteins was inhibited in PC9/IL-8 cells (Fig. 2d). These results provide the first evidence that introduction of IL-8 into gefitinib-sensitive lung cancer cells protects cells against gefitinib-induced apoptosis.

### Suppression of IL-8 enhanced gefitinib-induced cell death in EGFR TKI-resistant cells

**Figure 3:**
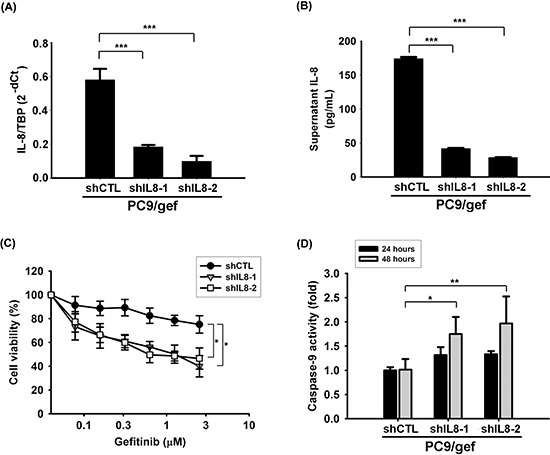
Knockdown IL-8 increased gefitinib-induced apoptosis IL-8 mRNA **A.** and IL-8 secretion **B.** in PC9/gef-shCTL and PC9/gef-shIL8 cells were analyzed by RT-qPCR and ELISA, respectively. The bar graph represents the mean ± s.d. for *n* = 3 independent experiments (***p < 0.001). **C.** Cellular viability of PC9/gef-shCTL and PC9/gef-shIL8 cells was determined in the absence or presence of gefitinib for 72 hours by MTT assays. Herein, a two-fold serial dilution was used for the experiment resulting in concentration curves of gefitinib from 2.5 μM to 0.078 μM (*p < 0.05). **D.** Caspase-9 activity of these cells was analyzed by luminescent assay after treatment of gefitinib for 24 hours or 48 hours. Each condition was normalized to the corresponding vehicle-treated PC9/gef-shCTL group. The bar graph represents the mean ± s.d. for *n* = 3 independent experiments (*p < 0.05, **p < 0.01).

To investigate whether knockdown of IL-8 could result in increasing gefitinib sensitivity, small hairpin RNA (shRNA) against *IL-8* was used to knockdown IL-8 in PC9/gef, and we established two stable shIL8 cell lines with independent target sequences against *IL-8* (PC9/gef-shIL8–1 and PC9/gef-shIL8–2) (Supplementary Table S4). We showed that both PC9/gef-shIL8 cell lines expressed lower levels of IL-8 than the control cells (PC9/gef-shCTL) (Fig. 3a–b). Both PC9/gef-shIL8 cell lines were more sensitive to the gefitinib treatment than PC9/gef-shCTL cells (Fig. 3c). Gefitinib-induced caspase-9 activity was significantly increased in PC9/gef-shIL8 cells compared with PC9/gef-shCTL cells (Fig. 3d). Moreover, we showed that knockdown of IL-8 with small interfering RNA (siIL-8) also resulted in recovery of gefitinib-induced apoptosis in PC9/gef or HCC827/gef cells (Supplementary Fig. S3). Collectively, these results indicate that IL-8 plays a crucial role in gefitinib resistance.

### IL-8 increased stem cell-like characteristics in lung cancer cells

**Figure 4:**
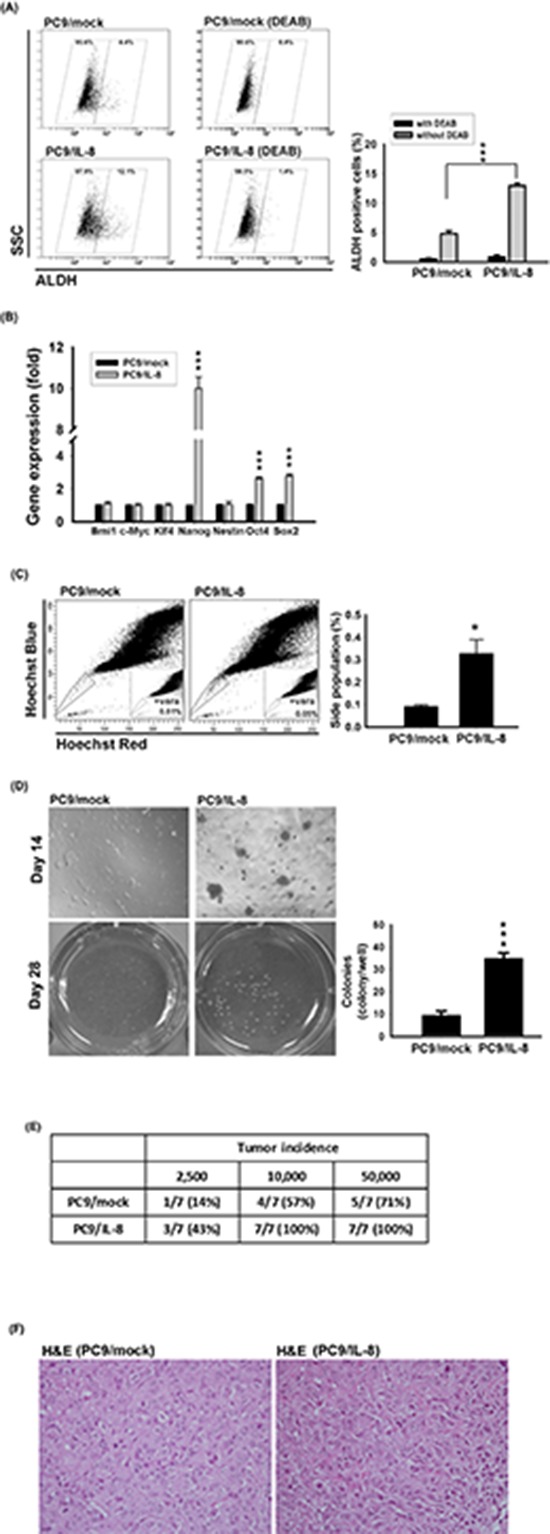
IL-8 conferred stem cell-like characteristics **A.** ALDH activity was examined by incubating PC9/mock and PC9/IL-8 cells with ALDH substrate in the presence and absence of DEAB. Plots show the results of three independent experiments (***p < 0.001). **B.** The levels of stem cell-related genes were analyzed in PC9/mock and PC9/IL-8 by RT-qPCR and normalized against that of TBP. The results shown represent the results of three independent experiments (***p < 0.001). **C.** Hoechst 33342 staining of PC9/mock and PC9/IL-8 cells. *Left*: Location of the side population in a representative experiment is indicated by gate and dot plots. *Right*: Quantification of results from four independent experiments (*p < 0.05). Verapamil (vera) was used to distinguish side population and non-side population as indicated. **D.** PC9/mock and PC9/IL-8 cells (2.5 × 10^3^ cells/well) were analyzed for colony formation in soft agar. *Left*: Photographs of colonies were taken on days 14 and 28. *Right*: Quantification of total colonies per well from three independent experiments (***p < 0.001). **E.** Tumor formation incidence. NOD.SCID mice were injected subcutaneously with the indicated numbers (2.5 × 10^3^ to 5 × 10^4^ cells) of PC9/mock and PC9/IL-8 cells (*n* = 7). Tumors were examined 10 weeks after injection. **F.** Hematoxylin-Eosin (H&E) staining of tumor from mice implanted with 2.5 × 10^3^ cells of PC9/mock or PC9/IL-8. Magnification is “400x”.

EMT is a biological process by which epithelial cells lose cell-cell adhesion, and have less E-cadherin expression. Both the EMT regulator (Slug) and IL-8 were up-regulated in PC9/gef cells and conferred resistance to gefitinib [4]. To investigate whether IL-8 initiated EMT to result in gefitinib resistance, we examined the expression of EMT-related genes. However, overexpression of IL-8 didn't induce EMT or up-regulation of EMT-related genes in PC9/IL-8 cells compared with PC9/mock cells (Supplementary Fig. S4). The EMT regulator (Slug) and IL-8 are involved in cell motility, and invasion in previous reports and this study, respectively [4]. Slug initiates cell invasion through repression of E-cadherin and activation of EMT, but IL-8 promotes cell motility by directly activating Rac GTPase (one member of Rho family) instead of regulating EMT mediators in previous studies [24, 25]. We previously showed that PC9/gef presented a higher proportion of ALDH-positive compared with PC9 cells and ALDH-positive cells in PC9 cells were resistant to gefitinib [18]. Here, we isolated ALDH-positive and ALDH-negative sub-populations from PC9/gef to determine the correlation between IL-8 expression and ALDH activity. Intriguingly, the ALDH-positive sub-population from PC9/gef showed simultaneously increased *IL-8* (Supplementary Fig. S5), supporting a positive correlation between IL-8 and stem-like characteristic. To investigate whether IL-8 contributes to stem-cell like activity, we found that the ALDH-positive cell population was increased in PC9/IL-8 cells compared with PC9/mock cells (Fig. 4a). A number of stem cell-associated genes, including *Nanog*, *Oct4*, and *Sox2*, were also significantly up-regulated in PC9/IL-8 cells (Fig. 4b); others, including *Bmi-1*, *c-Myc*, *Klf4* and *Nestin*, were not different.

Side populations have been shown to be enriched for cancer stem-like cells. Using Hoechst 33342 dye and verapamil to characterize the side population, we found a significant increase in the side population fraction in PC9/IL-8 cells compared to PC9/mock cells (0.33% vs. 0.09%) (Fig. 4c). Using another pair of cells, we found a significant increase of side population fraction in HCC827/IL-8 cells compared with HCC827/mock cells (1.86% vs. 0.53%) (Supplementary Fig. S6a–c). Furthermore, we performed colony-forming assays to investigate whether IL-8 drives the development of stem cell-like properties. Compared with PC9/mock cells, PC9/IL-8 cells gave rise to more and larger colonies in soft-agar cultures (Fig. 4d). We also found a significant increase of colonies in HCC827/IL-8 than HCC827/mock cells by clonogenic assay (Supplementary Fig. S6d), indicating IL-8 contributes to the inhibition of gefitinib-induced cell death and the development of stem cell-like characteristics. To further evaluate the effect of IL-8 on cell proliferation, we performed assays on growth rate, cell cycle and proliferation-related proteins (PCNA and cyclin D1). The cell proliferation rates of mock-infected and IL-8-infected cells were similar (Supplementary Fig. S7a). Moreover, over-expression of IL-8 didn't result in change of cell cycle and proliferation-related proteins (Supplementary Fig. S7b–c). Therefore, we excluded that IL-8 increased cell proliferation to lead to more colonies.

To determine the tumorigenicity *in vivo*, we subcutaneously injected cells into NOD.SICD mice and found that PC9/IL-8 cells gave rise to more visible tumors than PC9/mock cells (Fig. 4e). And, Hematoxylin-Eosin (H&E) stains of tumors from NOD.SCID mice injected with PC9/mock or PC9/IL-8 cells were shown in Fig. 4f. These results suggest that the IL-8-expressing cell population harbors a larger compartment of stem-like cancer cells with a higher potential for tumorigenicity.

### Inhibition of IL-8 reduced the stemness in EGFR TKI-resistant cells

**Figure 5:**
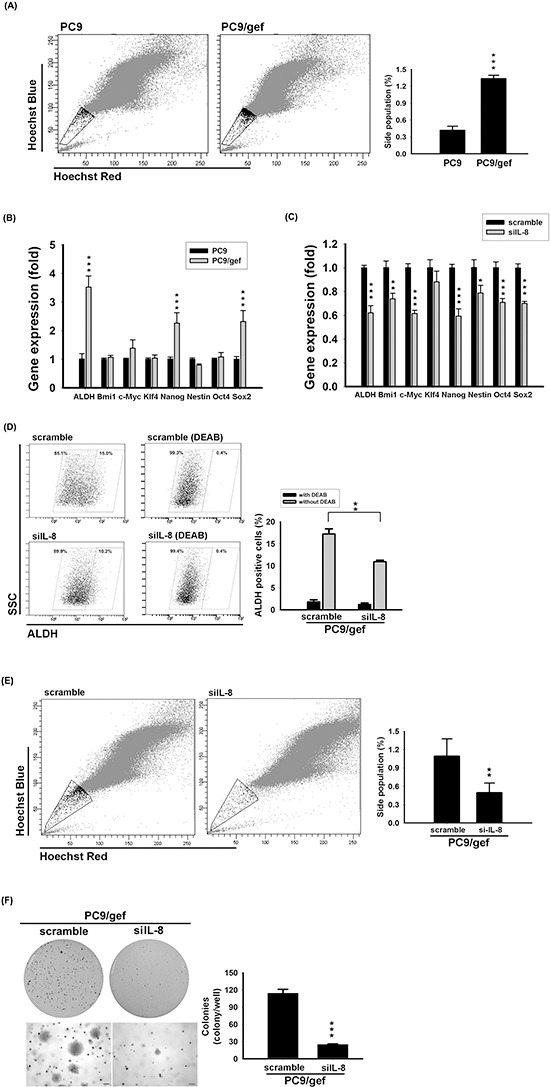
Knockdown IL-8 reduced stem cell-like characteristics **A.** Hoechst 33342 staining of PC9 and PC9/gef cells. *Left*: Location of the side population in a representative experiment is indicated by gate and dot plots. *Right*: Quantification of results represents the mean ± s.d. for *n* = 3 independent experiments. (***p < 0.001). **B.** The expression of stemness genes of PC9 and PC9/gef cells was quantified by RT-qPCR; the bar graph represents the mean ± s.d. for three determinations (***p < 0.001). **C.** Stem cell-related gene expressions in PC9/gef cells was examined by RT-qPCR after transient transfection with 50 nM IL-8 siRNA (siIL-8) for 48 hours. The bar graph represents the mean ± s.d. for three determinations (**p < 0.01, and ***p < 0.001). **D.** ALDH activity were examined by incubating with ALDH substrate in the presence or absence of DEAB after silencing IL-8 in PC9/gef cells. **E.** PC9/gef cells transfected with control (scramble) siRNA or siIL-8 were stained with Hoechst 33342 dye. *Left*: Representative experiment of four independent experiments shows the gate region, indicating the location of the side population, and dot plots. *Right*: Quantification of events collected for each sample (1 × 10^5^ cells). **F.** PC9/gef cells transfected with 50 nM scramble or siIL-8 cells (5 × 10^3^ cells/well) were analyzed for colony formation in soft agar. After 28 days, colonies were photographed and counted. All results are representative of three independent experiments (*p < 0.05, **p < 0.01, and ***p < 0.001).

We previously showed that PC9/gef cells presented a higher proportion of ALDH-positive cells than PC9 cells [18]. To identify more stemness-like abilities, we made an attempt to conduct side population analysis and investigate stemness-related gene expressions. Hoechst 33342 staining showed that PC9/gef displayed the presence of side population cells in 1.34% (Fig. 5a). Conversely, PC9 cells included less side population cells in 0.42% (Fig. 5a). Interestingly, HCC827/gef cells also displayed side population cells and more colonies than HCC827 cells (Supplementary Fig. S8a–b). We next investigated the expression of stemness-related genes between two pairs of EGFR TKI-sensitive and EGFR TKI-resistant cells. *ALDH1A1*, *Nanog*, and *Sox-2* were significantly up-regulated in PC9/gef (Fig. 5b). Similarly, up-regulation of stemness-related genes (*ALDH1A1*, *Nanog*, *Nestin*, *Bmi1*, and *Oct4*) was observed in HCC87/gef cells (Supplementary Fig. S8c), implying that these gefitinib-resistant cells contained a larger population of tumor-initiating cells. To determine whether IL-8 was involved in conferring stemness in PC9/gef and HCC827/gef cells, stemness-related gene expressions were evaluated after knockdown of IL-8. Notably, the stemness-related *ALDH1A1*, and *Nanog* were mostly suppressed in siIL-8 cells compared with control scrambled siRNA cells (Fig. 5c). Similarly, the ALDH activity was down-regulated in siIL-8 cells (Fig. 5d). Moreover, *IL-8* knockdown also reduced the side population fraction (1.10% *vs*. 0.50%) and diminished colonies in PC9/gef (Fig. 5e–f). The effects of knockdown of *IL-8* in HCC827/gef cells were also assessed. Likewise, knockdown of *IL-8* had most inhibitory potency on *ALDH1A1*, *Nanog*, *Bmi-1*, and *Sox-2* in HCC827/gef cells (Supplementary Fig. S8d). The side population fraction (1.15% vs. 0.52%) and colonies in long-term clonogenicity assay were both suppressed in HCC827/gef after knockdown of *IL-8* (Supplementary Fig. S8e–f). These results indicated that IL-8 is critical in regulating stem cell-like characteristics and gefitinib resistance in lung cancer cells.

### IL-8 conferred resistance to chemotherapy

Since IL-8 induced stem-like properties, we suggested that IL-8 confers resistance to traditional chemotherapy (such as paclitaxol). Cell viability assays were performed to evaluate the paclitaxol-induced apoptosis between PC9/mock and PC9/IL-8 cells. Both the percentage of AnnexinV-positive cells and paclitaxol-induced cell death were inhibited in PC9/IL-8 cells (Supplementary Fig. S9a–b). Moreover, we made an attempt to suppress IL-8 activity using neutralizing IL-8 antibody and investigated whether it can promote paclitaxol-induced cell death. We showed that neutralizing IL-8 antibody enhanced paclitaxol-induced cell death in PC9/gef cells, which suggested IL-8 still conferred resistance to traditional chemotherapeutics (Supplementary Fig. S9c–d).

### Effect of exogenous IL-8 on stemness and EGFR-TKI resistance

**Figure 6:**
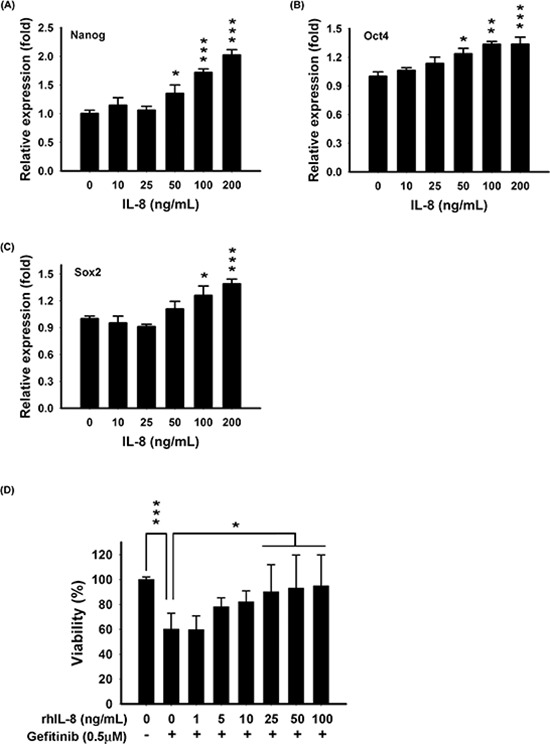
Exogenous IL-8 induced stemness and decreased EGFR TKI-induced cell death in PC9 cells Stem cell-related genes- Nanog **A.**, Oct4 **B.**, and Sox2 **C.** were examined after treatment with various concentrations of rhIL-8 (0–200 ng/mL) for 12 hours by RT-qPCR. The bar graph represents the mean ± s.d. for three determinations (*p < 0.05, **p < 0.01, and ***p < 0.001). **D.** PC9 cells were treated with various concentrations of rhIL-8 (0–100 ng/mL) for 24 hours, and then incubated with 0.5 μM gefitinib for another 24 hours. Cell viability was measured by MTT assays. The bar graph represents the mean ± s.d. for *n* = 3 independent experiments (*p < 0.05, and ***p < 0.001).

To investigate whether exogenous IL-8 contributes to stemness and EGFR-TKI resistance, recombinant human IL-8 (rhIL-8) was used. Treatment with rhIL-8 significantly increased *Nanog*, *Oct4*, and *Sox2* mRNA in PC9 cells, as determined by RT-qPCR (Fig. 6a–c). This result was consistent with the observation in Fig. 4b, as we showed that *Nanog*, *Oct4*, and *Sox2* were up-regulated in PC9/IL-8 cells. Further, we evaluated whether exogenous rhIL-8 could confer resistance against gefitinib. PC9 cells were cultured with various concentrations of rhIL-8 for one day, and then exposed to gefitinib for one day. As shown in Fig. 6d, IL-8-treated PC9 cells displayed resistance to gefitinib. This result indicates that exogenous rhIL-8 exhibits same function as endogenous IL-8 does.

## DISCUSSION

EGFR-TKIs have been widely used in treating non-small cell lung cancers with EGFR mutation-positive [26]. In the present study, IL-8 is highly associated with the cellular stemness and EGFR TKI resistance in lung cancer cells. We demonstrated that IL-8 elicits a protective effect against gefitinib-induced apoptosis and caspase activation. Knockdown of IL-8 circumvented EGFR TKI resistance and promoted gefitinib-induced apoptosis. Delineating the function of IL-8 in EGFR TKI resistance, we showed that IL-8 is capable of driving stem cell-like characteristics in lung cancer cells. Etopic introduction of IL-8 resulted in increase of side population, a greater potential for anchorage-independent growth, higher ALDH activity, and *in vivo* tumorigenic activity. In contrast, IL-8 inhibition in gefitinib-resistant cells diminished stem cell-like characteristics, and increased gefitinib-induced apoptosis. Moreover, high plasma IL-8 was associated with poor-progression-free survival in EGFR mutation-positive NSCLC patients receiving EGFR TKI as first-line treatment.

In the study, we identified that several chemokines were up-regulated in gefitinib-resistant cells including CXCL-1, CXCL-2, CXCL-6, and IL-8 (Table 1). Accumulating evidences have revealed that chemokines promote cancer cell growth and are associated with treatment resistance [25, 27]. Stromal cell-derived factor-1 is associated with tumor recurrence [28], and CXCL-1/2 mediate the chemoresistance in lung cancer [8]. Likewise, the role of IL-8 in mediating cancer cell migration, proliferation, and chemoresistance in an autocrine or paracrine manners has been demonstrated previously [27, 29]. Elevated IL-8 levels have been demonstrated in several type of cancers, and was correlated with survival or relapse [30–33]. Previous reports showed that the serum levels of IL-8 in lung cancer patients were in the pg/mL range [34–36], which is similar to our study. Previous studies also demonstrated that the level of IL-8 in cell supernatant (100 pg/mL) is consistent with serum level of IL-8, such as HCT116 cells [27, 36]. Furthermore, the level of IL-8 in cell line culture medium depended on cell type and absolute cell number [33, 36]. In this study, we showed the level of IL-8 was consistent with previous reports.

Increased tumorigenic activity, and re-activation of survival signaling including PI3K/Akt, and Ras/Raf/MAPK by IL-8 stimulation are demonstrated to confer resistance against chemotherapy [6, 29]. We showed that Akt is hyper-phosphorylated in IL-8-overexpressing cells. The PI3K/Akt pathway is one of the principal downstream effectors of IL-8-dependent, CXCR1/2-mediated signaling, leading to tumor progression [37]. Activation of the CXCR1/2 signaling by IL-8 leads to activation of NF-κB, forming a positive feedback loop further promotes tumor development [38]. In contrast, shutdown of IL-8 signaling via siRNA or neutralizing antibodies leads to sensitizes cancer cells to chemotherapy [39]. IL-8 was previously supposed to transactivate EGFR signaling in NSCLC [40]. Here, we showed the first time that IL-8 plasma level in NSCLC patients is correlated with duration of EGFR TKI treatment in NSCLC (Fig. 1d), and IL-8 intervenes in resistance of EGFR TKIs (Figs. 2, 3, and Supplementary Fig. S3).

As a notable finding, soluble cytokines and chemokines may regulate stem cell-like characteristics, and emerging evidence has shown that CSCs are responsible for tumor growth and metastasis, and contribute to chemotherapy resistance and relapse. CSC's marker expression is a critical element to distinguish cancer cells into stem-like or non-stem-like cells. Side population, ALDH activity and Naong expression have been recognized as hallmarks of lung stem cell markers [41]. Knockdown of IL-8 resulted in inhibition of *ALDH1A1*, *Bmi1*, *Nanog*, *Sox2* (> 30% reduction) both in PC9/gef and HCC827/gef cells (Fig. 5c and Supplementary Fig. 8d). However, we observed that *Bmi-1* and *Nestin* were up-regulated only in HCC827/gef cells, but not in PC9/gef cells (Fig. 5b and Supplementary Fig. 8c). It indicated that stemness-related genes may be differently regulated by IL-8 in PC9/gef and HCC827/gef cells. Previous studies showed that IL-8 and its cognate receptors, CXCR1/CXCR2, played an important role in CSC activity and chemoresistance. Recently, several studies indicated that specific blockade of CXCR1 or CXCR2 with neutralizing antibodies resulted in different inhibition of CSC population and cancer cell growth [11, 36]. It was suggested that the effect of IL-8 was mediated by CXCR1 and CXCR2, but the selection of receptor for IL-8 binding and actions were in a cell type-specific manner. In our study, we showed that PC9 cells are more abundant of CXCR1 than HCC827 cells (Supplementary Fig. S1). We suggest that the differential expression of CXCR1 and CXCR2 in these cell lines (PC9, and HCC827) may have different influence on CSC activity and other actions under IL-8 stimulation or knockdown of IL-8.

Besides IL-8, IGF1R (insulin-like growth factor 1 receptor) pathway was involved in gefitinib-resistance and stemness in gefitinib-resistant PC9 and HCC827 cells [42]. Activation of IGF1-IGF1R axis increased several stem cell markers (such as CD133, *ALDH1A1*, *Oct4*, *Nanog*, *CXCR4*, and *Sox2*) both in PC9/gef and HCC827/gef cells [42]. In our microarray database, we didn't observe up-regulation of IGF1 in PC9/gef cells. We suggested that different culture conditions and methods (step-wise escalation or high-concentration of gefitinib) used to establish gefitinib-resistant cell lines activate different pathways and stemness-related genes [42]. It should be concerned that the manner of EGFR TKI exposure influences the change of genotype and phenotype in EGFR TKI-resistant cells.

Studies have revealed that IL-8 secretion were up-regulated under chemotherapy, and IL-8 increased cancer stem cell populations [43, 44]. Moreover, IL-8 receptor, CXCR1, is also specially expressed in an ALDH-positive cancer cell sub-population and these cells are resistant to FASL-induced apoptosis [11]. Blockade of the CXCR1 evoked FasL-mediated apoptosis in a CSC sub-population. It suggests that the IL-8/CXCR-1 axis is associated with stem cell renewal and setmness-related sphere formation is diminished upon IL-8 inhibition [11, 45]. Interestingly, lung cancer cells surviving treatment with chemotherapeutic drugs in SCID mice propagated with CSCs-like characteristics and expressed elevated levels of CXCR1/2 [46]. Consistent with these findings, we showed that PC9/IL-8 cells have greater tumorigenesis potential in NOD.SCID mice and possess a larger fraction of side population cells compared to PC9/mock cells. We further found that CXCR1/2 was up-regulated in gefitinib-resistant cells, which would enhance the sensitivity to IL-8-mediated signal activation.

IL-8 expression was shown to be enriched in the side population of human melanoma cells which are capable of excluding anticancer drugs [47]. Side population cells are abundant of ATP-binding cassette (ABC) transporters [48]. Study reported that side population cells showed a great potential against EGFR TKI-induced apoptosis [19], and we consistently found ABCB1 transporter is up-regulated in EGFR TKI-resistant cells (data not shown). However, it is still unclear whether EGFR TKIs are substrates of ABC transporters, and the association between EGFR TKI resistance and ABC transporters is controversial.

In conclusion, our data suggest that diminishing the CSC sub-population through depletion of IL-8 in combination with gefitinib administration might potentially overcome EGFR-TKI resistance.

## METHODS

### Cell lines

We established the gefitinib-resistant lung adenocarcinoma cell lines- PC9/gef and HCC827/gef from gefitinib-sensitive cells- PC9 and HCC827, respectively [4, 20]. PC9/IL-8, HCC827/IL-8 and PC9/gef-shIL8 cells were generated by stable lentiviral infection with the *IL-8* gene in PC9, HCC827 cells or shRNA against *IL-8* in PC9/gef cells. These cell lines were authenticated using short tandem repeat profiling. All cell lines were cultured in RPMI-1640 supplemented with 10% fetal bovine serum (FBS) and propagated at 37°C in a humidified 5% CO_2_ incubator.

### Oligonucleotide microarray

Messenger RNA (mRNA) expression profiles of PC9 and PC9/gef were generated using Affymetrix Human Genome U133 plus 2.0 GeneChips (Affymetrix; Santa Clara, CA), which contain 54,675 probe sets capable of analyzing the expression levels of 47,400 transcripts and variants. Oligonucleotide cDNA microarray analyses were performed by the Integrated Core Facility for Functional Genomics of National Science Council in Taiwan according to the manufacturer's protocols (Santa Clara, CA, http://www.affymetrix.com/). The expression profiles were deposited in the GEO database (GSE60189).

### Real-time quantitative reverse transcription-PCR (RT-qPCR)

Complementary DNA (cDNA) was generated using a High-Capacity cDNA reverse transcription kit (Applied Biosystems; Foster city, CA). Gene specific primer sequences are listed in Supplementary Table S1. Quantitative-PCR was carried out on an ABI 7500 system. Relative mRNA expression levels were calculated using the 2^−ΔCt^ method, where ΔCt = (sample Ct – TBP Ct). It was considered “undetectable” if the value of Ct > 40.

### Enzyme-linked immunosorbent assay (ELISA)

Cells were plated at a density of 5 × 10^3^ cells/well in a 24-well plate. After cells had adhered, the culture medium was replaced with fresh medium and plates were incubated for 48 hours. Supernatants were collected for quantification of IL-8 protein using a Quantikine IL-8 ELISA kit (R&D Systems; Minneapolis, MN).

### Patient population and study design

All treatment-naive patients with stage IV lung adenocarcinoma diagnosed between October 2010 and October 2013 were identified. Only those with *EGFR* mutation-positive and who received EGFR-TKIs as their first-line treatment were investigated in this study. Plasma IL-8 level in patients was determined by IL-8 ELISA kit and this study was approved by the Institutional Review Board of National Taiwan University Hospital.

### Apoptosis assays

Apoptosis was detected using an Annexin-V-FITC detection kit (BD Biosciences). Briefly, cells were incubated with fluorescein isothiocyanate (FITC)-conjugated Annexin-V and propidium iodide (PI) for 15 minutes in the dark. Analyses were performed using flow cytometry and CXP analysis software (Beckman Coulter, CA).

### Anchorage-independent growth

Cells were suspended in top agar (0.35% agarose) and plated in triplicate onto bottom layers of 0.5% agar in a 6-well plate. The cells were fed 2 mL of medium every 5–7 days. Colonies were photographed after 4 weeks and analyzed using Image J software.

### Determination of tumorigenicity in NOD.SCID mice

Animal experiments were approved by the Institutional Animal Care and Use Committee (IACUC No.20110492) of the National Taiwan University, Taiwan. NOD.CB17-*Prkdc^scid^*/NcrCrlBltw (NOD.SCID) male mice (BioLASCO; Taipei, Taiwan) at five weeks old were transplanted with cells. Briefly, cancer cells were suspended in HBSS buffer at density of 5 × 10^5^ cells/mL, and subcutaneously injected into each mouse. All mice were examined regularly for the development of tumors, and cells were scored as tumorigenic if a palpable nodule appeared at the site of injection within 10 weeks and increased in size.

### ALDH activity

The ALDEFLUOR kit (Stemcell Technologies; Vancouver, Canada) was used to identify cell populations with ALDH enzymatic activity. Cells were suspended in ALDEFLUOR Assay Buffer containing ALDH substrate. Following a 30 minute incubation at 37°C and centrifugation, the cells were re-suspended for analysis using a FACSAria flow cytometer (BD Biosciences). Samples treated with the inhibitor, diethylaminobenzaldehyde (DEAB), were used as negative controls to gate the ALDH-negative region.

### Side population

Cells were stained with 5 μg/mL Hoechst 33342 (Sigma-Aldrich; St. Louis, MO) at 37°C for 90 minutes. PI staining was used to isolate dead cells, and 150 μM verapamil (Sigma-Aldrich) was used to distinguish side population. Side population cells were sorted from the main cell population using a FACSAria through services provided by the Cell Sorting Core Facility.

### Caspase-9 activity

Cells were re-suspended with density of 5 × 10^4^ cells/mL and hung in 96-Well Hanging Drop Plates (Perfecta3D; Ann Arbor, MI) for 48 hours, and then incubated with gefitinib. After incubation, caspase-9 activity was measured with the caspase-Glo 9 luminescence kit (Promega; Madison, WI). Briefly, the cellular lysates were incubated with substrates to generate luminescent signal and the luminescence was recorded.

### Statistical analysis

Student's *t*-test was used for comparing means of continuous variables between two groups. Progression-free-survival curves were plotted using the Kaplan-Meier method and compared the differences between the groups using the log-rank test. Two sided *p*-values < 0.05 were considered statistically significant.

## SUPPLEMENTARY DATA, FIGURES AND TABLES


