# Supplementary material for: IL-8 confers resistance to EGFR inhibitors by inducing stem cell properties in lung cancer
Source: Oncotarget. 2015 Mar 18;6(12):10415–31. doi: 10.18632/oncotarget.3389 (PMC4496364; doi:10.18632/oncotarget.3389)
Supplement: Supplementary file 1 [file oncotarget-06-10415-s001.pdf]

SUPPLEMENTARY DATA

Reagents

Primary antibodies against caspase-3, full-length caspase-9, cleavage caspase-9, PARP, phospho-Akt, and Akt were purchased from Cell Signaling Technology (Danvers, MA). β-actin and α-tubulin were purchased from Millipore (Darmstadt, Germany). Lamin B2 was purchased from Genetex (Irvine, CA). Complementary DNA (cDNA) plasmid for *IL-8* (IMAGE 3882471) was purchased from the Bioresource Collection and Research Center (Hsinchu, Taiwan), and small hairpin RNA against *IL-8* (TRCN0000232050 and TRCN0000232051) and lentivirus packaging vectors were purchased from Academia Sinica (Taipei, Taiwan). Hoechst 33342 was purchased from Thermo Fisher Scientific (Waltham, MA). Human recombinant IL-8 peptide was purchased from Peprotech (Rocky Hill, NJ).

Oligonucleotide microarray analysis

GeneChips were read using the Affymetrix GeneChip scanner 3000 7G, and raw data were processed using the GC-RMA algorithm and analyzed with GeneSpring GX software (Silicon Genetics; Redwood City, CA). Differentially expressed genes, defined as those with greater than a 2-fold change in expression level, were subjected to biological and pathway analyses using the bioinformatics resource, DAVID (Database for Annotation, Visualization and Integrated Discovery, http://david.abcc.ncifcrf.gov/), to identify over-represented biological responses and molecular pathways for each gene.

Construction of stable clones

PC9/IL-8 and HCC827/IL-8 cells were generated by stable lentiviral infection of PC9 and HCC827 cells with the full-length *IL-8* gene. Following lentivirus-mediated gene transfer, stably infected cells were selected by incubating at a low density with G418 (500 μg/mL) for 14 days, and then were maintained in media containing 200 μg/mL G418. PC9/gef-shIL-8 cells were generated by stable lentiviral infection of PC9/gef cells with shIL-8, and selected by incubating at a low density with puromycin (2 μg/mL) for 14 days, and then were maintained in media containing 1 μg/mL puromycin.

Invasion assay

Transwells were coated with Matrigel (BD Biosciences; Franklin Lakes, NJ) for invasion assays, and 5% FBS was used as chemoattractant. After 18 hours of incubation, cells remaining on the top of the insert were removed and then the inserts were fixed with 70% ethanol and stained with 0.5% crystal violet. Total invasive cells on the lower surface of the insert were counted at 200x magnification.

Gene knockdown

Gene knockdown was achieved by transfecting cells with a pool of four IL-8 − specific siRNA duplexes (Catalog #L-004756–00, Dharmacon; Pittsburgh, PA) using Lipofectamine 2000 reagent (Invitrogen), as described by the manufacturer.

MTT assay

Cells were re-suspended with density of 5×10^4 cells/mL and hung in 96-Well Hanging Drop Plates (Perfecta3D; Ann Arbor, MI) for 48 hours as manufacturer's instructions, and then incubated with gefitinib. After 72 hours, cells were incubated MTT (3-(4, 5-cimethylthiazol-2-yl)-2, 5-diphenyl tetrazolium bromide) to form formazan. The product was dissolved in DMSO (Dimethyl sulfoxide) and the absorbance was recorded at 550 nm using a VICTOR^3 multiple reader (PerkinElmer; Waltham, MA).

Cell death ELISA

PC9/gef and HCC827/gef cells were transiently transfected with scramble or siRNA against IL-8 for 48 hours. After knockdown of IL-8, cells were seeded on 96-well plates (5×10^3 cells/well) and treated with vehicle or gefitinib for 24 hours. Mono- and oligo-nucleosomes in the cytoplasmic fraction were measured by the Cell Death Detection ELISA^{PLUS} Kit (Roche; Penzberg, Germany) according to the manufacturer's instructions. Briefly, the lysates were transferred into the wells coated with streptavidin, and incubated with anti-histone antibodies, horseradish peroxidase–conjugated anti-DNA antibodies, and then the substrate. Absorbance was measured at 405 nm.

Clonogenic assay

HCC827/mock and HCC827/IL-8 cells were plated at 200 cells per well and exposed to vehicle or gefitinib at indicated concentrations for 48 hours. The drugs were

then washed away, and the cells were allowed to grow for 14 days. After knockdown of IL-8, HCC827/gef cells were plated at 200 cells per well and exposed to gefitinib for 96 hours followed by incubation in a drug-free medium for another 10 days. The colonies were fixed and stained with 0.5% crystal violet and the experiments were repeated in quadruplicate.

Statistical analysis

All clinical analyses were performed using SPSS 15.0 statistical software (SPSS Inc., Chicago, IL, USA). Student's *t*-test was used for comparing means of continuous variables between two groups. Two-sided *p*-values < 0.05 were considered statistically significant.

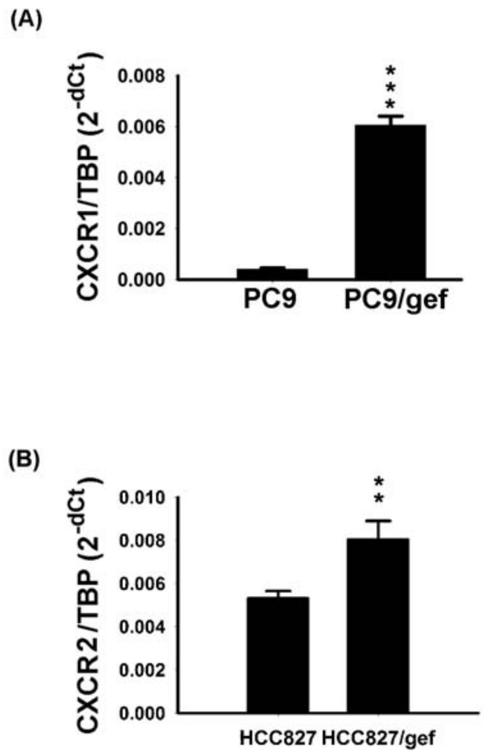

Supplementary Figure S1: CXCR1 and CXCR2 expression in PC9, PC9/gef, HCC827, and HCC827/gef cells. Expression of CXCR1 and CXCR2 mRNA was detected by RT-qPCR in **A.** PC9, PC9/gef, and **B.** HCC827, HCC827/gef cells. TBP was used as an internal control for normalization (***$p < 0.001$).

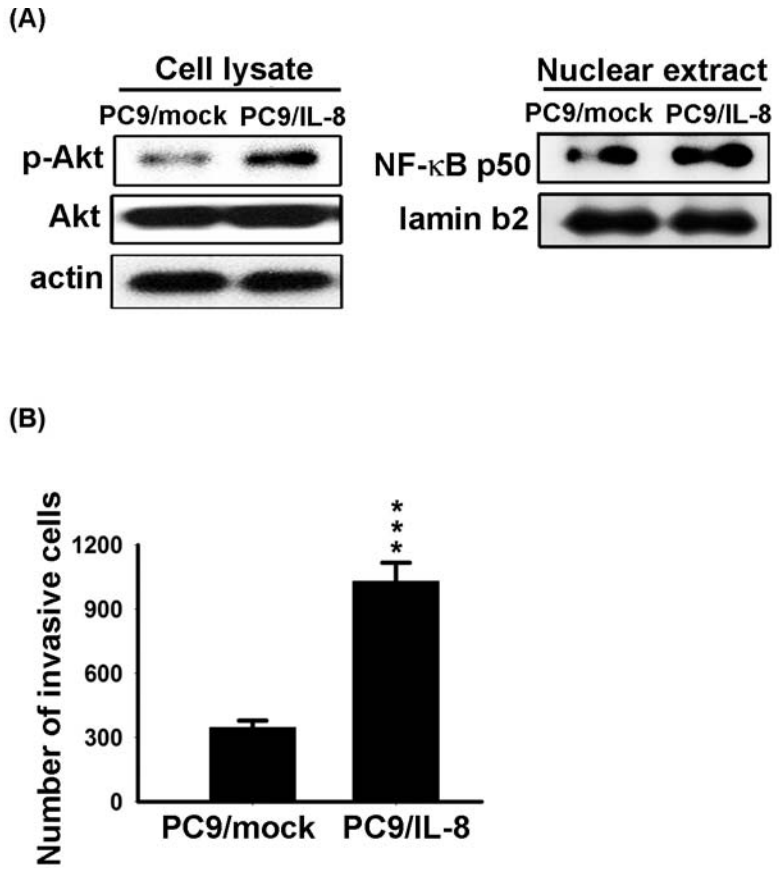

Supplementary Figure S2: Activation of IL-8 downstream pathway. A. Whole-cell extracts and nuclear extracts from PC9/mock and PC9/IL-8 cells were collected and analyzed by SDS-PAGE and Western blotting for the detection of Akt, phospho-Akt, and NF-κB p50. Actin and lamin B2 were used as loading controls for the whole-cell compartment and nuclear fraction, respectively. **B.** Invasive capability of PC9/mock and PC9/IL-8 cells was evaluated by transwell invasion assays. Quantification of migrated cell numbers was based on four independent wells (***$p < 0.001$).

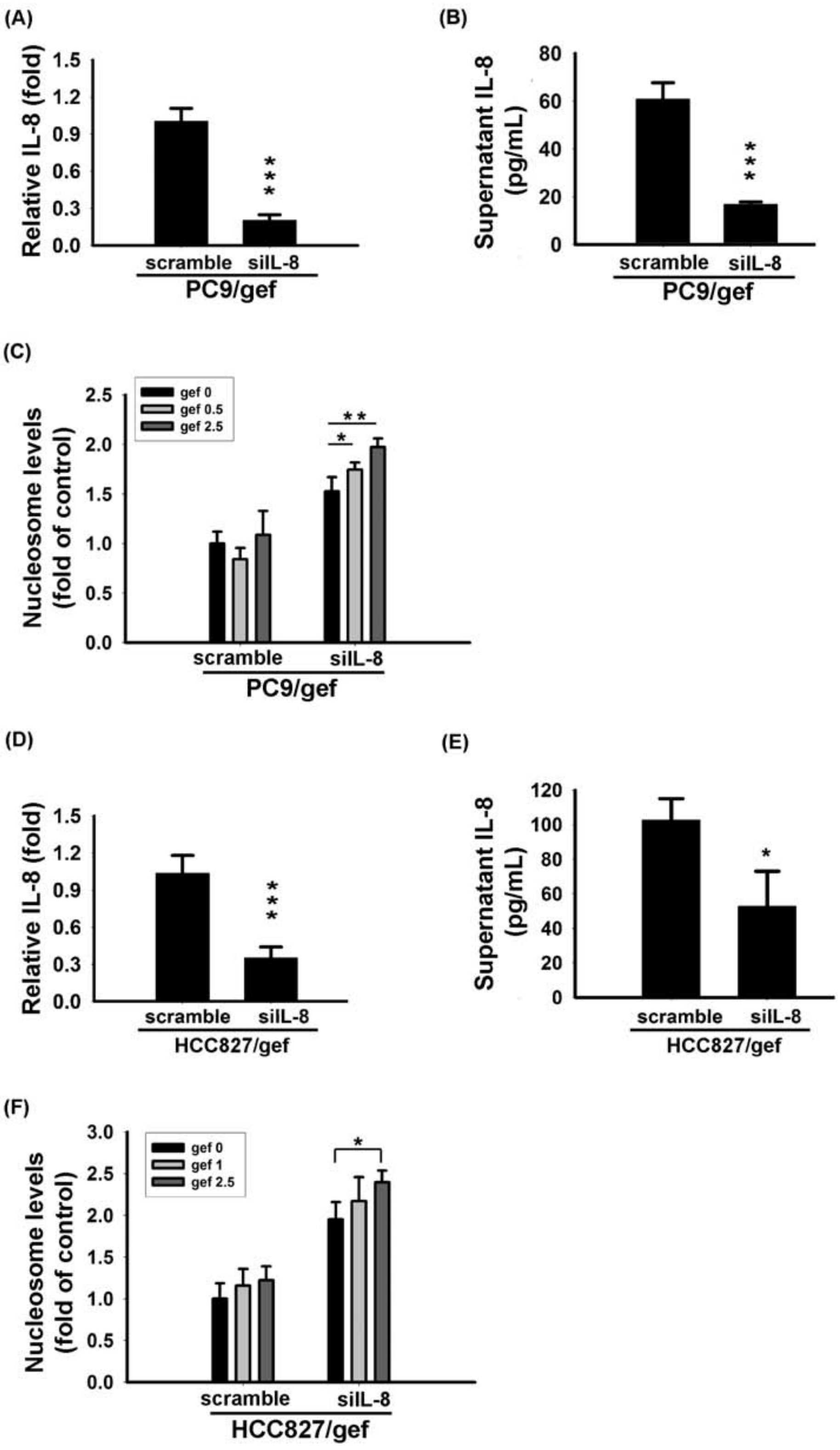

Supplementary Figure S3: Knockdown of IL-8 increased gefitinib-induced apoptosis. IL-8 mRNA **A, D.** and protein level **B, E.** were evaluated in PC9/gef and HCC827/gef cells after transient transfection with control siRNA (scramble) or IL-8 siRNA (siIL-8; 50 nM for PC9/gef, and 100 nM for HCC827/gef) for 48 hours. The bar graph represents the mean ± s.d. for $n = 3$ independent experiments (*$p < 0.05$). **C, F.** After knockdown of IL-8, apoptotic effect in PC9/gef and HCC827/gef cells was determined in the absence or presence of gefitinib for 24 hours with cell death ELISA as described in “Supplementary Methods”. The bar graph represents the mean ± s.d. for $n = 3$ independent experiments (*$p < 0.05$, **$p < 0.01$).

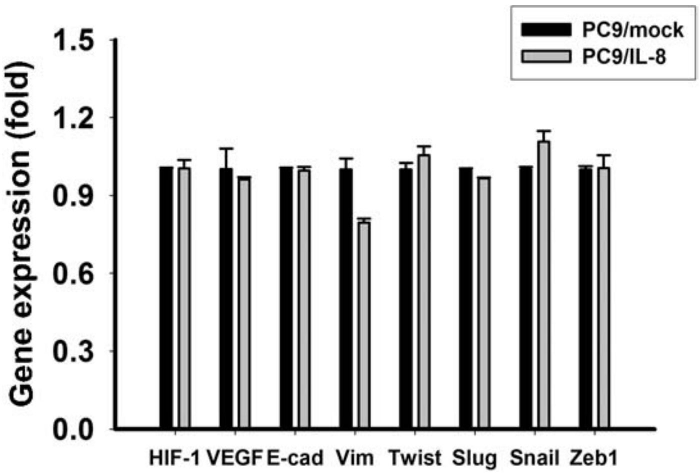

Supplementary Figure S4: Up-regulation of IL-8 didn't change the expression of EMT-related genes. Expressions of E-cadherin (E-cad), vimentin (Vim), and EMT-related genes (HIF-1, VEGF, twist, slug, and zeb1) were determined in PC9/mock and PC9/IL-8 using RT-qPCR.

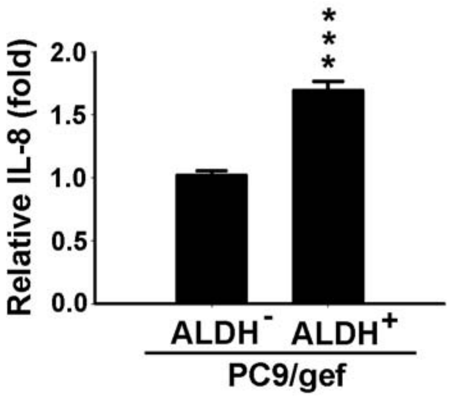

Supplementary Figure S5: ALDH-positive sub-population showed higher IL-8 expression. PC9/gef cells were incubated with ALDH substrates and sorted by flow cytometry. After isolation of ALDH-positive sub-population from PC9/gef cells, IL-8 mRNA was determined using SYBR-based RT-qPCR. TBP was used as an internal control for normalization. The bar graph represents mean ± s.d. for five determinations. ***$p < 0.001$ compared with ALDH-negative sub-population.

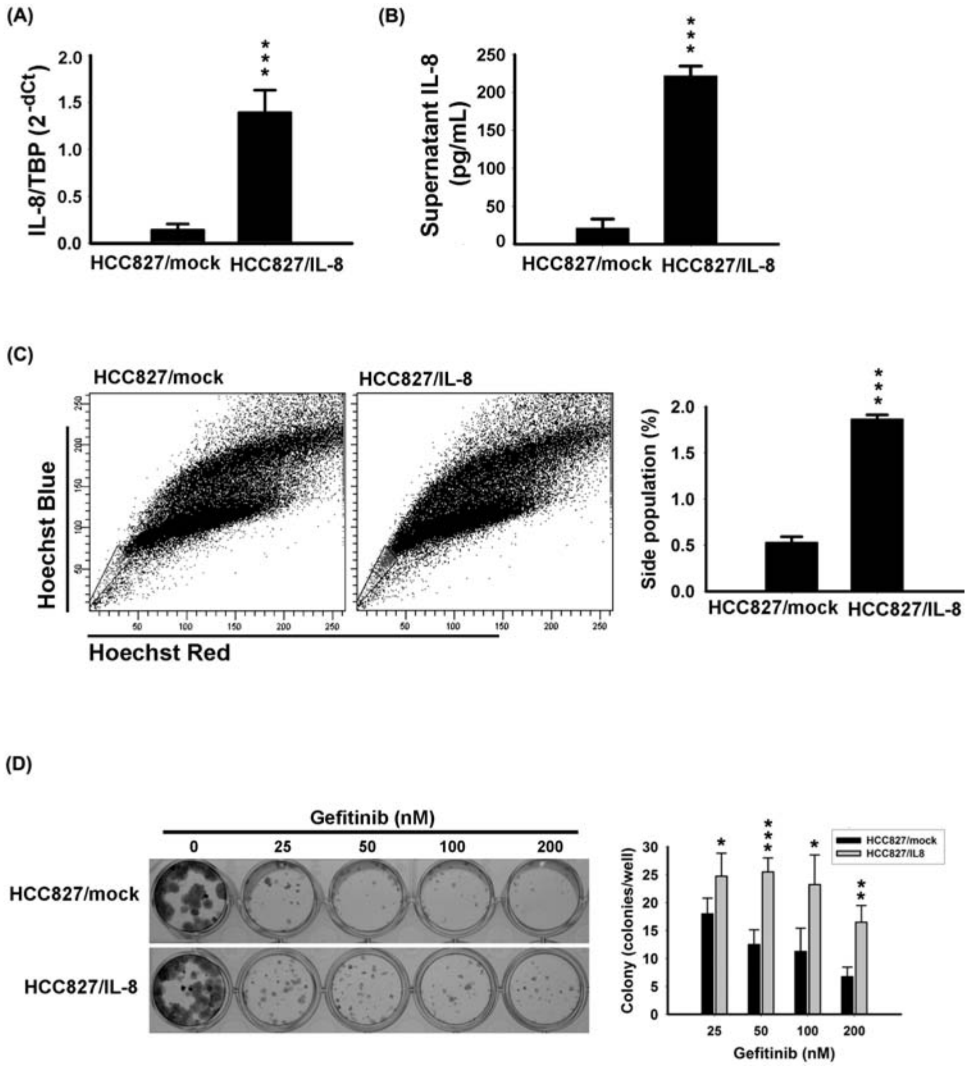

Supplementary Figure S6: IL-8 conferred stem cell-like characteristics on HCC827 cells. IL-8 expression in stable HCC827/mock and HCC827/IL-8 cell lines was evaluated by RT-qPCR **A.** and IL-8 ELISA **B. C.** Hoechst 33342 staining of HCC827/mock and HCC827/IL-8 cells. *Left*: Location of the side population in a representative experiment is indicated by gate and dot plots. *Right*: Quantification of results from five determinations (***p < 0.001). **D.** Clonogenic capacity of HCC827/mock and HCC827/IL-8 cells was assessed. After treatment of gefitinib, HCC827/mock and HCC827/IL-8 were incubated in drug-free medium after another 14 days as described in “Supplementary Methods”.

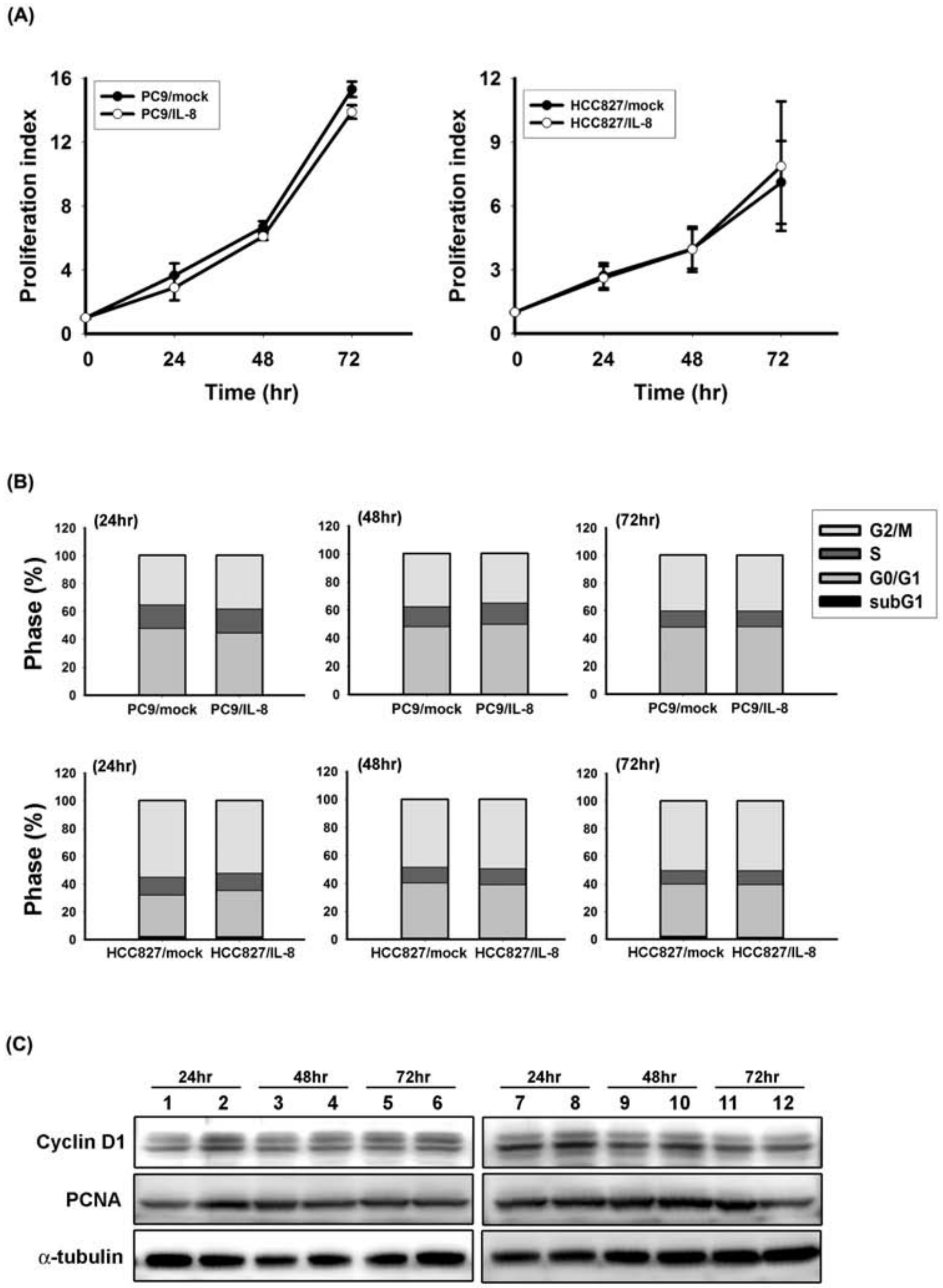

Supplementary Figure S7: IL-8 didn't enhance cell proliferation. Proliferation rate **A.** and cell cycle **B.** were examined in PC9/mock, PC9/IL-8, HCC827/mock, and HCC827/IL-8 cells at the time point of 24, 48, and 72 hours by MTT and flow cytometry assays. Quantification of results represent the mean ± s.d. for $n = 3$ independent experiments. **C.** Cell lysates were collected at the time point of 24, 48, and 72 hour, and proliferation-associated proteins (PCNA and cyclin D1) were analyzed by western blots. (Lane 1, 3, 5 represent PC9/mock; Lane 2, 4, 6 represent PC9/IL-8; Lane 7, 9, 11 represent HCC827/mock; Lane 8, 10, 12 represent HCC827/IL-8). Data are representative of two independent experiments.

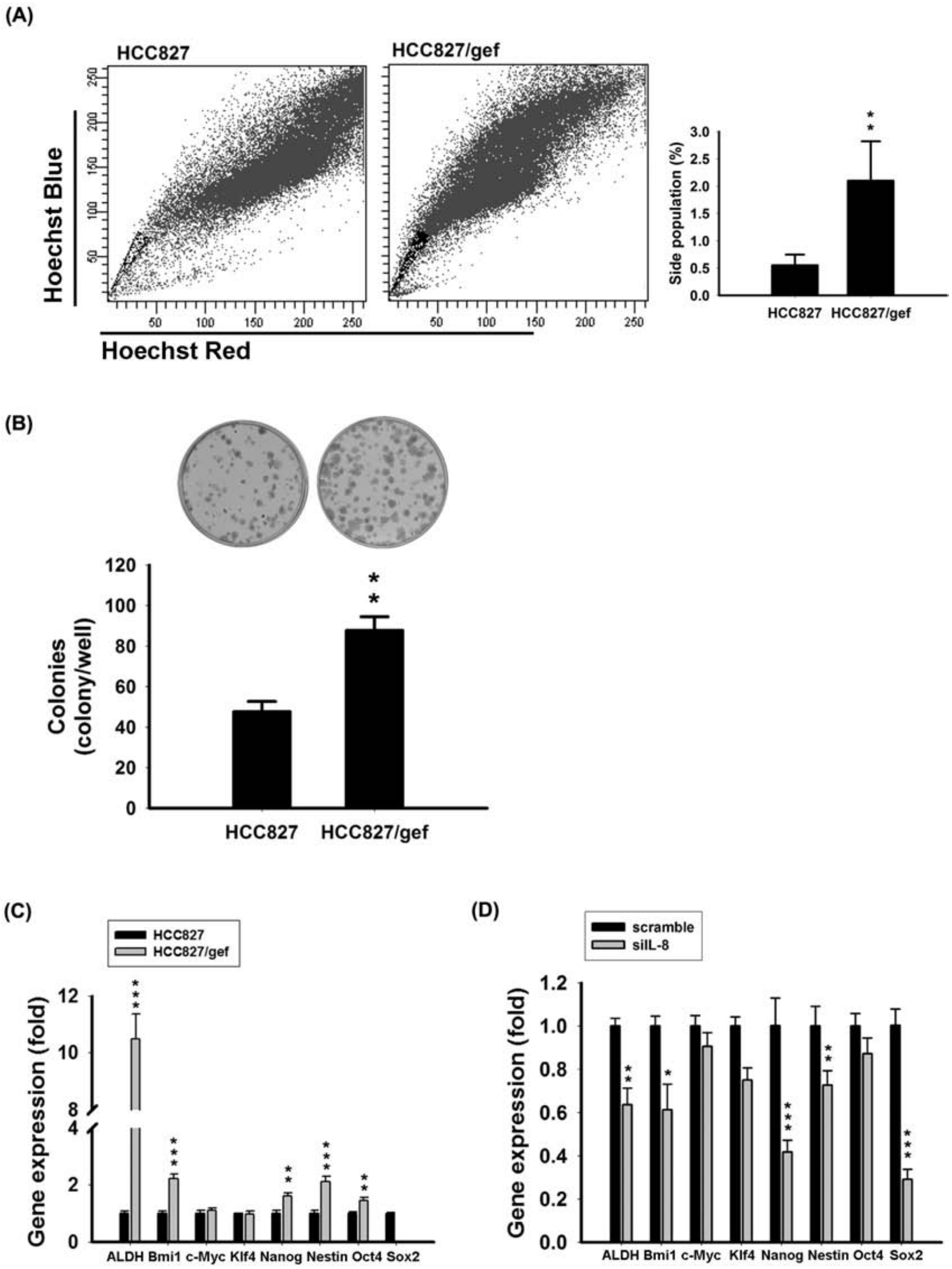

Supplementary Figure S8: Knockdown of IL-8 reduced stem cell-like characteristics and clonogenic capacity. A. Hoechst 33342 staining of HCC827 and HCC827/gef cells. *Left*: Location of the side population in a representative experiment is indicated by gate and dot plots. *Right*: Quantification of results represent the mean ± s.d. for $n = 3$ independent experiments (**$p < 0.01$). **B.** Clonogenic capacity of HCC827 and HCC827/gef cells was assessed. HCC827 and HCC827/gef (400 cells/well) were incubated in medium for 14 days, and the colonies were fixed, and stained with 0.5% crystal violet. The experiment was repeated in triplicate. **C.** The expression of stemness-related genes from HCC827 and HCC827/gef cells was quantified by RT-qPCR; the bar graph represents the mean ± s.d. for three determinations (**$p < 0.01$, and ***$p < 0.001$). **D.** Stemness-related genes were analyzed by RT-qPCR after silencing IL-8 in HCC827/gef cells. The bar graph is representative of six determinations (*$p < 0.05$, **$p < 0.01$, and ***$p < 0.001$).

(*Continued*)

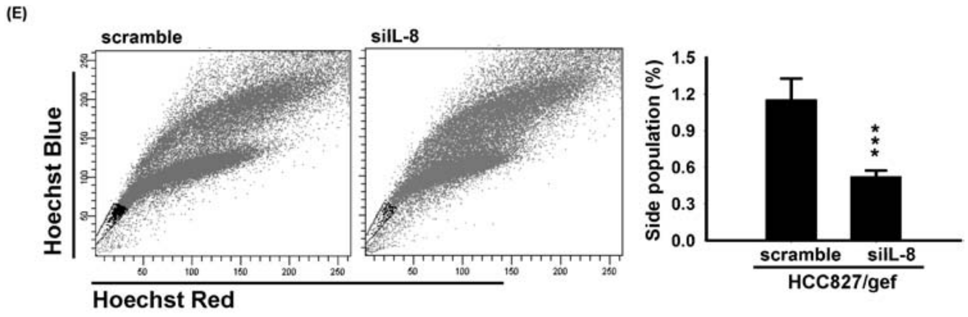

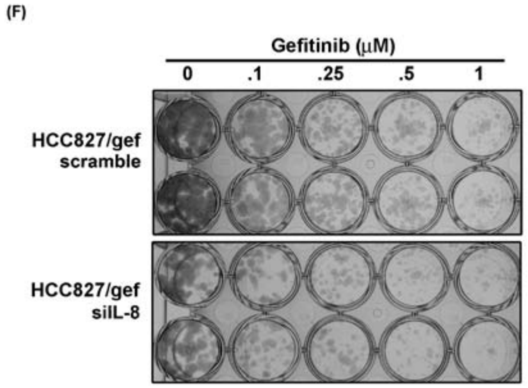

Supplementary Figure S8 (*Continued*): E. HCC827/gef cells transfected with control (scramble) siRNA or siIL-8 were stained with Hoechst 33342 dye. *Left*: Representative experiment of five determinations shows the gate region, indicating the location of the side population, and dot plots. *Right*: Quantification of events collected for each sample (1×10^5 cells). **F.** Effect of IL-8 on clonogenic capacity was assessed by silencing IL-8 in HCC827/gef cells. Both scramble- or siIL-8-transfected cells were treated with gefitinib for 96 hours, and then incubated in drug-free medium after another 10 days as described in "Supplementary Methods".

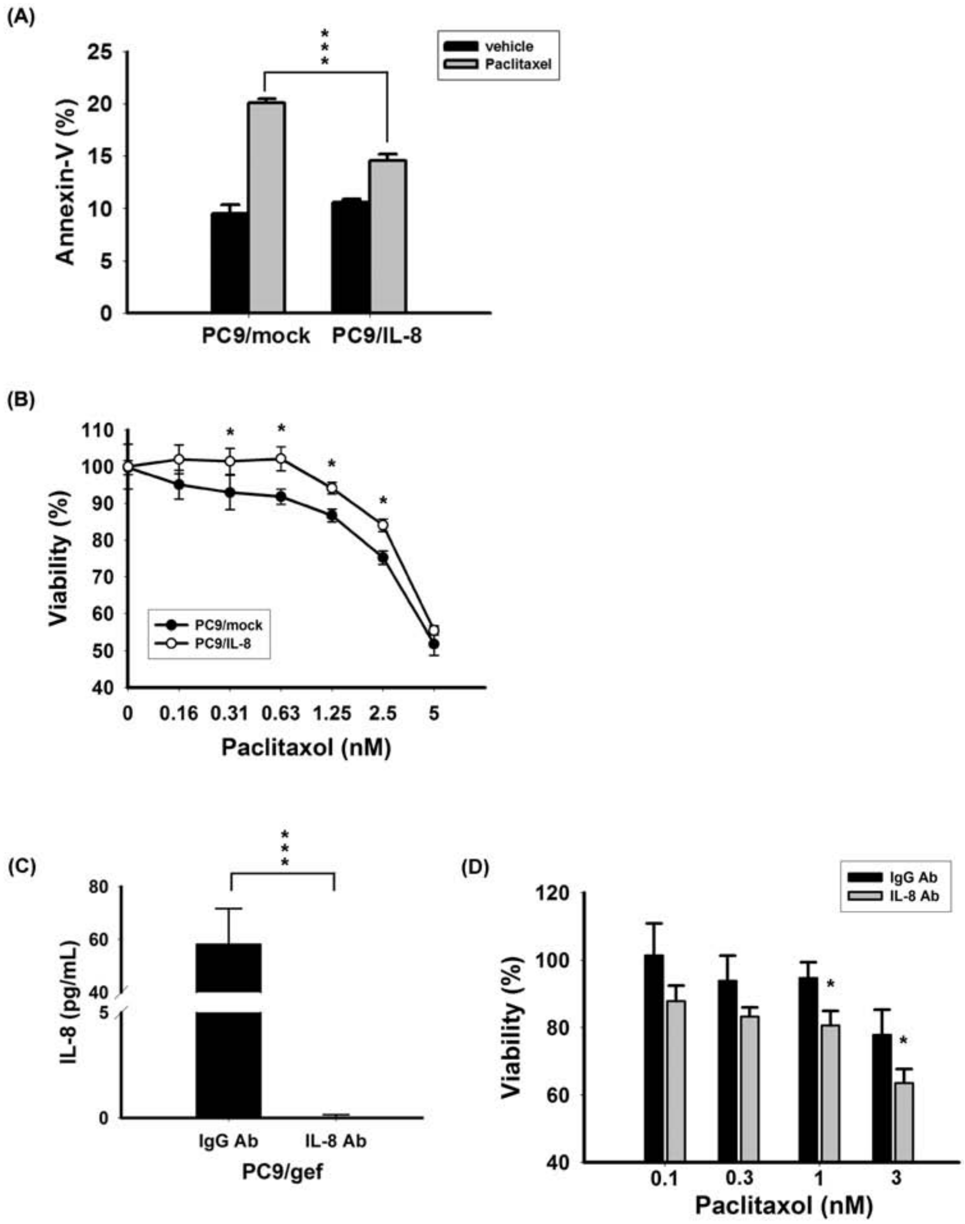

Supplementary Figure S9: IL-8 conferred resistance to chemotherapeutics. **A.** After 24 hours of treatment with 3 nM paclitaxol, the percentage of apoptotic cells was evaluated in PC9/mock and PC9/IL-8 cells by Annexin-V staining. The bar graph represents the mean ± s.d. for $n = 3$ independent experiments (***$p < 0.001$). **B.** Cellular viability of PC9/mock and PC9/IL-8 cells was determined in the absence or presence of paclitaxol for 48 hours by MTT assays. Herein, a two-fold serial dilution was used for the experiment resulting in concentration curves of paclitaxol from 5 nM to 0.16 nM (*$p < 0.05$). **C.** IL-8 protein level was evaluated in PC9/gef cells after incubation with neutralizing IL-8 antibody for 24 hours. The bar graph represents the mean ± s.d. for three determinations (***$p < 0.001$). **D.** PC9/gef cells were treated with indicated concentrations of paclitaxol and 0.1 μg/mL neutralizing IL-8 antibody for 48 hours, and cell viability was determined by MTT assays. The bar graph represents the mean ± s.d. for three determinations (*$p < 0.05$).

Supplementary Table S1. List of primers

Gene	Primer
IL-8-F	5′-ACTCCAAACCTTTCCACCC-3′
IL-8-R	5′-AAACTTCTCCACAACCTCTG-3′
IL-8 probe	5′-TGTGCACAGGAGCCAAGAGTGAAGA-3′
TBP-F	5′-CACGAACCACGGCACTGATT-3′
TBP-R	5′-TTTTCTTGCTGCCAGTCTGGAC-3′
TBP probe	5′-TGTGCACAGGAGCCAAGAGTGAAGA-3′
IL-1A-F	5′-GGTTGAGTTTAAGCCAATCCA-3′
IL-1A-R	5′-TGCTGACCTAGGCTTGATGA-3′
IL-1B-F	5′-TACCTGTCCTGCGTGTTGAA-3′
IL-1B-R	5′-TCTTTGGGTAATTTTTGGGATCT-3′
IL-6-F	5′-TTCAATGAGGAGACTTGCCTG-3′
IL-6-R	5′-ACAACAACAATCTGAGGTGCC-3′
CXCR1-F	5′-GCCGGTGCTGCAGTTAGATCA-3′
CXCR1-R	5′-ATCTTCATCTGCAGGTGGCAT-3′
CXCR2-F	5′-CAGCGACCCAGTCAGGATTT-3′
CXCR2-R	5′-TCCCAGCAGGCTCAGCAG-3′
ALDH1A1-F	5′-TCGTCTGCTGCTGGCGACAA-3′
ALDH1A1-R	5′-AGCCCAACCTGCACAGTAGCG-3′
Bmi1-F	5′-AAATGCTGGAGAACTGGAAAG-3′
Bmi1-R	5′-CTGTGGATGAGGAGACTGC-3′
c-Myc-F	5′-GGAACGAGCTAAAACGGAGCT-3′
c-Myc-R	5′-GGCCTTTTCATTGTTTTCCAACT-3′
Nanog-F	5′-ATTCAGGACAGCCCTGATTCTTC-3′
Nanog-R	5′-TTTTTGCGACACTCTTCTCTGC-3′
Nestin-F	5′-AGGAGGAGTTGGGTTCTG-3′
Nestin-R	5′-GGAGTGGAGTCTGGAAGG-3′
Klf4-F	5′-CCGCTCCATTACCAAGAGCT-3′
Klf4-R	5′-ATCGTCTTCCCCTCTTTGGC-3′
Sox2-F	5′-CGAGTGGAAACTTTTGTCGGA-3′
Sox2-R	5′-TGTGCAGCGCTCGCAG-3′
Oct4-F	5′-GTGGAGAGCAACTCCGATG-3′
Oct4-R	5′-TGCTCCAGCTTCTCCTTCTC-3′

Supplementary Table S2. Functional annotation using the online DAVID bioinformatics resource

Category	Term	Counts	*P*-Value
KEGG pathway	Cytokine-cytokine receptor interaction	23	2.2×10^{-4}
KEGG pathway	Pathways in cancer	19	5.8×10^{-2}
KEGG pathway	MAPK signaling pathway	16	6.8×10^{-2}
KEGG pathway	Cell-adhesion molecules	13	3.3×10^{-3}
KEGG pathway	JAK-STAT signaling pathway	12	2.8×10^{-2}
KEGG pathway	Graft-versus-host disease	9	6.9×10^{-5}
KEGG pathway	Type I diabetes mellitus	8	7.6×10^{-4}
KEGG pathway	Antigen processing and presentation	8	3.3×10^{-2}
KEGG pathway	NOD-like receptor signaling pathway	7	2.6×10^{-2}
KEGG pathway	Viral myocarditis	7	4.6×10^{-2}
KEGG pathway	Small cell lung cancer	7	$8. \times 10^{-2}$
KEGG pathway	Hematopoietic cell lineage	7	9.7×10^{-2}
KEGG pathway	Allograft rejection	6	9.7×10^{-2}
KEGG pathway	Autoimmune thyroid disease	6	3.9×10^{-2}
KEGG pathway	mTOR signaling pathway	6	4.2×10^{-2}
KEGG pathway	Prion disease	5	3.9×10^{-2}

Supplementary Table S3. EGFR mutation status in lung adenocarcinoma patients

EGFR status	Patient number
Del in exon 19	37
L858R	33
G719A	2
L861Q	2
delE709-T710 insD	1
Total	75

Supplementary Table S4. The shRNA sequences against IL-8

Gene	Sequence
shIL8-1	5′-CCGGTTGGCAGCCTTCCTGATTTCTCTCGAGAGAAATCAGGAAGGCTGCCAATTTTTG-3′
shIL8-2	5′-CCGGACTTAGATGTCAGTGCATAAACTCGAGTTTATGCACTGACATCTAAGTTTTTTG-3′
